# The Impact of Increased Homozygosity on Human Fertility: A Comprehensive Review

**DOI:** 10.7759/cureus.49000

**Published:** 2023-11-18

**Authors:** Pranjal Prem, Komal N Muneshwar, Suyash Agrawal, Arpita Jaiswal

**Affiliations:** 1 Community Medicine, Jawaharlal Nehru Medical College, Datta Meghe Institute of Higher Education and Research, Wardha, IND; 2 Medicine, Jawaharlal Nehru Medical College, Datta Meghe Institute of Higher Education and Research, Wardha, IND; 3 Obstetrics and Gynaecology, Jawaharlal Nehru Medical College, Datta Meghe Institute of Higher Education and Research, Wardha, IND

**Keywords:** genetic diversity, recessive genetic disorders, consanguineous marriages, genetic counseling, fertility, homozygosity

## Abstract

This comprehensive review explores the multifaceted relationship between increased homozygosity and human fertility, delving into the genetic, ethical, cultural, and public health dimensions of this complex phenomenon. Homozygosity, characterized by identical alleles at specific gene loci, can result from consanguineous marriages, genetic drift, and population isolation. The review highlights key findings, including the heightened risk of recessive genetic disorders, the implications for immune system diversity, and the influence on complex traits and diseases. It underscores the critical role of genetic counseling in addressing these consequences, considering the ethical implications, and respecting cultural practices. The delicate balance between genetic diversity and cultural norms is emphasized, calling for increased awareness and community engagement. Looking ahead, the review suggests emerging technologies, longitudinal studies, and interdisciplinary research as crucial avenues for further exploration, with the ultimate goal of informing effective public health policies and interventions that safeguard genetic diversity and cultural traditions for future generations.

## Introduction and background

Homozygosity is a genetic state in which an individual possesses two identical alleles at a particular locus on a pair of homologous chromosomes. In simpler terms, an individual inherits the same gene variant from both parents for a specific trait. This genetic condition contrasts with heterozygosity, where an individual carries two different alleles for the same gene [1]. Homozygosity can result from genetic mechanisms, including inheritance from closely related family members (consanguinity) and the random assortment of alleles due to genetic drift or geographic isolation. Understanding the implications of increased homozygosity on human fertility is a complex and multifaceted topic that warrants a comprehensive review [2].

Human fertility is a fundamental aspect of the survival and growth of populations and the perpetuation of genetic diversity. It encompasses the ability of individuals and couples to reproduce and have offspring. Maintaining healthy fertility rates is crucial for the sustainability of a population. Factors affecting fertility can have significant consequences for both individuals and society as a whole [3]. A multitude of biological, environmental, and sociocultural factors influence fertility. Genetic factors, including homozygosity, can play a role in determining an individual's reproductive success and the health of offspring. Understanding how homozygosity affects human fertility is essential for addressing questions related to genetic diversity, reproductive health, and the prevalence of inherited genetic disorders [4].

The purpose of this comprehensive review is to delve into the intricate relationship between increased homozygosity and human fertility. This review aims to provide a thorough examination of the genetic and biological aspects of homozygosity, its causes, and its implications for reproductive health. It will also explore the impact of homozygosity on pregnancy outcomes and congenital disabilities. Furthermore, the review will assess the existing body of research, population studies, and relevant data to shed light on the connection between homozygosity and fertility rates in different populations and geographic regions. Ethical and cultural dimensions of this topic will also be considered, as they play a critical role in understanding the broader implications and potential interventions.

## Review

Understanding homozygosity

Definition and Types of Homozygosity

Simple homozygosity: Simple homozygosity is a genetic state where an individual possesses two identical alleles at a specific gene locus. These alleles can represent either a dominant or a recessive trait. In this case, both alleles are identical, but it's important to note that this condition does not necessarily imply the presence of a genetic disorder or disease. Simple homozygosity can occur naturally in populations with no significant consanguinity or shared ancestry. It is a common genetic state and can result from the random assortment of alleles during reproduction [5].

Homozygosity by descent: Homozygosity by descent occurs when an individual inherits two identical alleles from a common ancestor. This phenomenon is often observed in consanguineous populations, where individuals are more likely to share a recent common ancestor due to intermarriage within the family or community. As a result, the alleles in question are passed down through generations from the same ancestor. This type of homozygosity is more likely to be associated with a higher risk of inheriting recessive genetic disorders because of the shared ancestry and the potential to inherit two disease-causing alleles [2].

Homozygosity by state: Homozygosity by state refers to the situation where an individual possesses two identical alleles purely by chance and not because of shared ancestry. This type of homozygosity can occur in populations due to the effects of genetic drift. Genetic drift is a random process where the frequency of alleles changes over generations due to chance events rather than selection. In such cases, the frequency of alleles can become more homogenous, leading to the occurrence of homozygosity by state. This type of homozygosity is essential in understanding how genetic diversity can be influenced by random events in small and isolated populations, even when there is no significant shared ancestry [6].

Genetic Mechanisms Leading to Homozygosity

Consanguineous marriages: Consanguineous marriages involve individuals who marry close relatives, such as first cousins or even more closely related family members. These unions are a direct and pronounced cause of increased homozygosity within a population. When individuals who share a recent common ancestor procreate, their offspring have a higher likelihood of inheriting identical alleles for specific genes. The shared ancestry leads to a greater probability of homozygosity, which can be associated with beneficial and detrimental effects. On the one hand, it may preserve certain advantageous traits within a family. Still, on the other hand, it significantly increases the risk of inheriting two copies of harmful recessive alleles, which can result in the manifestation of genetic disorders [7].

Genetic drift: Genetic drift is a mechanism that can lead to increased homozygosity in small and isolated populations. It is a process of random fluctuations in allele frequencies over time, which occurs due to chance events rather than natural selection. In small populations, random events can have a pronounced impact on the genetic makeup of the population. This can result in an accumulation of homozygous individuals for specific genes as the frequency of particular alleles increases purely by chance. Genetic drift can lead to the fixation of specific alleles, making them the only variant in the population, ultimately increasing homozygosity [8].

Population isolation: Geographical and social isolation of populations can contribute to increased homozygosity. When populations are isolated from one another, whether due to geographic barriers or social factors, the gene flow between them is limited. This means new genetic variation from external sources is less likely to be introduced into the population. Over time, as the population reproduces internally and does not exchange genes with other populations, specific alleles may become more common, leading to increased homozygosity. This is particularly significant in island populations or culturally isolated groups, where limited interbreeding with outsiders can lead to higher levels of homozygosity [9].

Consequences of Homozygosity in the Genome

Increased risk of recessive genetic disorders: Homozygosity significantly heightens the risk of expressing recessive genetic disorders. In populations with increased levels of homozygosity, individuals are more likely to inherit two identical copies of a disease-causing allele from both parents. This is especially prevalent in consanguineous populations where shared ancestry leads to the transmission of similar alleles. As a result, the probability of offspring inheriting two copies of a detrimental recessive allele is notably elevated, making it more likely for these alleles to be expressed. The expression of two copies of a disease-causing allele can lead to the manifestation of the corresponding genetic disorder, increasing the prevalence of these conditions in consanguineous communities [10].

Impact on the immune system: Homozygosity can have a substantial impact on the diversity of the immune system. A diverse array of genes associated with the immune response is crucial for effectively combating various pathogens and infections. When homozygosity leads to a lack of genetic diversity within these immune-related genes, the immune system's ability to recognize and defend against various pathogens may be compromised. Consequently, individuals with reduced immune diversity may become more susceptible to infections, as their immune systems lack the necessary genetic variation to mount effective defenses against a broader spectrum of pathogens. This can be particularly relevant in populations with high levels of homozygosity, where the immune system's reduced diversity may translate into an increased vulnerability to various infectious diseases [11].

Influence on complex traits and diseases: Homozygosity's impact extends beyond Mendelian genetic disorders and can also influence complex traits and diseases that involve the interaction of multiple genes. In populations with increased levels of homozygosity, the reduced genetic diversity can affect the heritability and expression of complex traits such as height, intelligence, or susceptibility to certain diseases. Additionally, homozygosity can influence the occurrence and progression of complex diseases, including autoimmune conditions and certain types of cancer. This is because these conditions often result from a combination of genetic factors, and reduced genetic diversity can alter the risk and severity of such diseases. Thus, homozygosity's influence is not limited to single-gene disorders but extends to more intricate traits and diseases with a multifactorial genetic basis [12-15].

Genetic implications of increased homozygosity

Increased Likelihood of Recessive Genetic Disorders

Recessive alleles: Recessive genetic disorders often necessitate the inheritance of two copies of a mutant allele, one from each parent, for the disease to manifest. In populations with elevated levels of homozygosity, the frequency of individuals who carry two identical copies of the same recessive allele significantly increases. This increase in homozygosity raises the likelihood of individuals inheriting two copies of a recessive mutant allele for a specific gene, making it more probable that these alleles will come together in offspring. As a result, the frequency of homozygous individuals for the disease-causing allele rises, amplifying the risk of the disorder manifesting in the population [16].

Disease prevalence: The increased likelihood of homozygosity in consanguineous populations is directly linked to a higher prevalence of specific recessive disorders, such as cystic fibrosis or sickle cell anemia. When consanguinity is expected in a population, the genetic relatedness among individuals is more significant, leading to a higher probability of both carriers and affected individuals in the community. This elevated prevalence of recessive disorders has substantial implications for public health, healthcare systems, and genetic counseling. It places an increased burden on healthcare resources and necessitates the development of specific healthcare strategies for the early diagnosis and management of these disorders. Genetic counseling becomes especially important in such populations to help individuals and couples make informed decisions about family planning and the potential risks associated with consanguinity [2].

Genetic load: The genetic load theory proposes that increased homozygosity can result in a higher burden of deleterious alleles within a population, potentially affecting overall health and well-being. The genetic load refers to the cumulative effect of carrying and potentially expressing harmful alleles. In populations with heightened levels of homozygosity, there is an increased likelihood of individuals carrying two identical copies of deleterious alleles, which can contribute to a higher genetic load. This genetic burden can impact the population's health, potentially reducing overall fitness, increasing disease susceptibility, and impacting reproductive success. Understanding the concept of genetic load is vital for assessing the implications of homozygosity on human health and the well-being of populations, particularly in consanguineous or isolated communities where genetic diversity is limited [17].

Impact on the Immune System

Limited immune diversity: Homozygosity can result in a reduced diversity of immune-related genes within an individual's genome. The immune system relies on a diverse array of genes to recognize and combat a wide range of pathogens, including bacteria, viruses, and other microorganisms. When an individual is homozygous for specific immune-related genes, they possess identical alleles for those genes. This genetic uniformity can limit the ability of the immune system to recognize and respond to a broad spectrum of pathogens effectively. As a result, the diversity of immune responses may be constrained, potentially compromising the individual's ability to defend against a broad array of infectious agents [18].

Susceptibility to infections: Individuals with limited immune diversity due to homozygosity may be more susceptible to certain infections and diseases. The reduced genetic variability in immune-related genes can hinder the immune system's capacity to mount an effective defense against a variety of pathogens. This increased susceptibility is particularly relevant in populations with high levels of homozygosity, where genetic diversity is naturally limited. Such individuals may experience a higher likelihood of contracting infectious diseases. They may face challenges mounting a robust immune response to these pathogens, potentially leading to more severe or prolonged infections [19].

Autoimmune diseases: While increased homozygosity can influence susceptibility to infections, it can also impact the risk of developing autoimmune diseases. Autoimmune diseases occur when the immune system mistakenly targets and attacks the body's tissues. These conditions are complex and often result from genetic and environmental factors. In populations with elevated homozygosity, there may be a lack of genetic diversity in immune-related genes, making it more likely for specific autoimmune-prone genetic variants to be expressed. This can increase the risk of developing autoimmune diseases, as the immune system's responses may become dysregulated due to the limited genetic diversity, potentially leading to erroneous self-tissue targeting. Thus, homozygosity can influence not only susceptibility to infections but also the risk of developing autoimmune conditions, highlighting the broad impact of genetic diversity on immune-related health outcomes [20].

Influence on Complex Traits and Diseases

Polygenic traits: Complex traits such as height, intelligence, and susceptibility to diseases like diabetes or cardiovascular conditions often result from the interplay of multiple genes. Reduced genetic diversity due to increased homozygosity can influence the heritability and expression of these polygenic traits. When homozygosity is more prevalent in a population, certain combinations of genetic variants occur more frequently, potentially affecting the distribution of trait-related alleles. This can lead to variations in the prevalence and expression of complex traits, as the limited genetic diversity may skew the genetic contributions to these traits. In some cases, this can result in the increased prevalence of specific trait outcomes or the concentration of risk alleles for certain diseases, impacting the health and characteristics of the population [21].

Epistasis: Epistasis refers to the interactions between genes where the effect of one gene depends on the presence of other specific genes. Increased homozygosity can alter the patterns of epistatic interactions within a population. When individuals are homozygous for specific genes, the potential for epistasis may differ from a population with higher genetic diversity. This can lead to unexpected trait outcomes and disease risks. Certain genetic combinations that are rare in populations with more genetic diversity may become more common in populations with increased homozygosity, potentially influencing the heritability of traits and disease susceptibility in unique ways [22].

Population-specific traits: Populations with high levels of homozygosity may develop unique genetic traits and adaptations. Some of these traits may be beneficial in specific environmental contexts. Increased homozygosity can lead to the fixation of specific alleles, as shared ancestry and limited gene flow can reinforce specific genetic characteristics. Studying these population-specific traits can provide valuable insights into the complex interplay between homozygosity and genetic diversity. Understanding the genetic adaptations that have evolved in response to increased homozygosity can shed light on how populations adapt to their genetic constraints and the specific environmental pressures they face [23].

The relationship between homozygosity and fertility

Impact on Reproductive Health

Studies and Findings

Increased fertility challenges: Research has indicated that higher levels of homozygosity can be linked to increased challenges in achieving and maintaining pregnancies. These challenges may manifest as infertility or difficulties in conception. The genetic relatedness between partners in consanguineous marriages can elevate the risk of inheriting two copies of deleterious alleles, possibly leading to reproductive challenges. Such challenges can result from an increased likelihood of transmitting genetic disorders to offspring or from the genetic constraints imposed by shared ancestry. As a consequence, these populations may experience a higher prevalence of infertility or difficulties in conceiving, which can have significant implications for family planning and reproductive health [24].

Prenatal health risks: In consanguineous populations with elevated levels of homozygosity, there is an associated higher risk of adverse prenatal outcomes. These outcomes can include preterm births, low birth weight, and neonatal mortality. The shared genetic background of the parents in consanguineous unions can increase the likelihood of homozygosity for specific alleles associated with prenatal health risks. This can result in a more significant occurrence of adverse outcomes during pregnancy and early infancy, affecting both the health of the newborn and the well-being of the parents. These prenatal health risks necessitate closer monitoring and healthcare interventions to mitigate the potential negative consequences [25].

Consanguinity and reproductive health: The prevalence of consanguineous marriages in specific regions has been linked to higher rates of inherited genetic disorders, which, in turn, can significantly impact reproductive health. When consanguinity is expected in a population, there is an increased likelihood of offspring inheriting two copies of disease-causing alleles, raising the risk of genetic disorders. These disorders can affect not only the health of the offspring but also the reproductive health of the parents. The need for genetic counseling, carrier testing, and informed family planning becomes particularly important in such populations to address the potential risks associated with consanguinity and its impact on reproductive health [26].

Genetic Load Hypothesis

The genetic load hypothesis postulates that increased homozygosity within a population leads to a higher genetic load, a term referring to the cumulative burden of harmful alleles. This hypothesis suggests that populations with a higher genetic load may experience reduced reproductive success and overall fitness. Understanding the genetic load hypothesis is essential for comprehending the potential consequences of increased homozygosity on reproductive health and fertility [27].

Influence on pregnancy outcomes

Miscarriages and Stillbirths

Recessive disorders: In populations with increased levels of homozygosity, there is a heightened risk of offspring inheriting two identical copies of recessive disease-causing alleles from their parents. This occurrence can significantly impact pregnancy outcomes. When both parents are carriers of a recessive allele for a specific disorder, there is a one in four chance (25%) that their offspring will inherit two copies of the disease-causing allele. This double dose of the detrimental allele can lead to the expression of the genetic disorder, potentially resulting in developmental abnormalities that are severe and incompatible with life. In such cases, these developmental abnormalities can lead to miscarriages or stillbirths. These pregnancy outcomes represent a significant concern for couples who are carriers of recessive alleles in populations with elevated homozygosity [28].

Embryonic lethality: In some instances, homozygous combinations of specific alleles can result in embryonic lethality, which refers to the death of the developing embryo during the early stages of pregnancy. These lethal combinations prevent the embryo from progressing to a viable fetus, leading to spontaneous abortions or miscarriages in the earliest stages of pregnancy. The embryonic lethality can result from genetic incompatibility, where the combination of alleles leads to severe developmental defects incompatible with life. As a result, these pregnancies are often lost during the early stages, and the occurrence of embryonic lethality can be elevated in populations with increased homozygosity, particularly in regions with high rates of consanguinity [29].

Birth Defects

Increased expression of recessive alleles: Homozygosity, characterized by having two identical alleles at a specific gene locus, significantly elevates the likelihood that a recessive allele will be expressed. Recessive alleles are typically masked or suppressed in the presence of a dominant allele. However, when an individual is homozygous for a recessive allele, there is no dominant allele to mask its effects, which can lead to genetic conditions associated with physical or developmental abnormalities. In populations with heightened homozygosity, there is a greater likelihood that individuals will inherit two copies of a recessive disease-causing allele from their parents. As a result, these individuals may be at a higher risk of expressing these detrimental alleles and developing the associated genetic conditions [30].

Complex trait interaction: Congenital disabilities can sometimes result from the complex interaction of multiple genes. These defects may not be solely determined by a single gene but rather by the intricate interplay of various genes and their interactions. Homozygosity can influence the genetic diversity and interaction of these genes. In populations with elevated levels of homozygosity, the limited genetic diversity may alter the patterns of gene-gene interactions, potentially leading to the expression of specific trait combinations or the occurrence of congenital disabilities that are influenced by the synergy of multiple genes. This complexity can make it more challenging to predict and understand the genetic basis of specific congenital disabilities, highlighting the multifaceted nature of the impact of homozygosity on complex traits and the development of genetic conditions [31].

Insights from Population Studies

Variability in fertility: Populations with increased homozygosity may display variability in fertility rates among individuals. This variability can stem from the genetic diversity within the population and the distribution of homozygosity-inducing factors. Some individuals may experience reduced fertility due to the higher likelihood of inheriting two copies of detrimental alleles, which can result in genetic conditions that impact reproductive health. In contrast, others may maintain normal reproductive health, mainly if they do not inherit two copies of these detrimental alleles. The variability in fertility rates within such populations can have significant consequences for family planning, genetic counseling, and the overall reproductive health of the community [32].

Impact on population dynamics: Research on homogeneous populations characterized by elevated levels of homozygosity offers valuable insights into how homozygosity can influence population dynamics. This includes its impact on population growth and genetic diversity. In such populations, the limited genetic diversity can lead to specific genetic traits and adaptations that may influence the population's ability to thrive in particular environmental conditions. The study of these population dynamics provides a broader understanding of how increased homozygosity can shape the genetic makeup of a community and its capacity to adapt to environmental changes or challenges. It also offers insights into the potential consequences of sustained homozygosity on the long-term viability of the population [33].

Mechanisms for coping with homozygosity

Evolutionary Adaptations

Natural selection and genetic drift: Evolutionary processes like natural selection and genetic drift can play crucial roles in helping populations adapt to the challenges posed by increased homozygosity. Natural selection favors the survival and reproduction of individuals with advantageous traits or genetic variations. In populations with heightened homozygosity, specific genetic variants that offer adaptive advantages may become more prevalent over time. These advantageous alleles can reduce the negative impacts of homozygosity on overall fitness. Genetic drift, a random process influencing allele frequencies, can also lead to the fixation of specific alleles. While genetic drift can increase homozygosity, it can also lead to the fixation of beneficial alleles that enhance the population's fitness and health. Together, these evolutionary processes can help mitigate the effects of homozygosity by favoring adaptive genetic variations [34].

Balancing selection: In some cases, balancing selection can help maintain genetic diversity in specific loci, even in populations with consanguinity or genetic drift. Balancing selection occurs when different alleles are favored in different circumstances, preventing one allele from becoming fixed in the population. This diversity can provide a buffer against the adverse effects of increased homozygosity. Specific alleles may be advantageous in particular environments or under certain conditions, leading to the preservation of genetic diversity. Balancing selection can help ensure that the population retains genetic variation that may be essential for coping with changing environmental challenges, thereby mitigating the potential consequences of homozygosity [35].

Local adaptations: Populations exposed to specific environmental conditions may undergo local adaptations. These adaptations favor the survival and reproduction of individuals with particular genotypes well-suited to their environment. In such cases, the effects of homozygosity may be mitigated as individuals with locally adaptive traits thrive and reproduce. These adaptations can provide a mechanism for populations to overcome the challenges associated with increased homozygosity in specific contexts. By favoring individuals with advantageous genotypes in their particular environment, local adaptations can enhance the overall fitness and resilience of the population [36].

Role of Genetic Counseling

Genetic education and counseling: Genetic counseling is pivotal in addressing the potential consequences of increased homozygosity, particularly in populations with high consanguinity rates. Genetic counselors are well-equipped to provide information and support to individuals and couples regarding their genetic risks. They can inform couples about their risk of transmitting recessive genetic disorders to their offspring, helping them make informed decisions about family planning. Through education and counseling, individuals can better understand the implications of their genetic backgrounds, enabling them to make choices that align with their reproductive goals and values [37].

Carrier testing: In populations with elevated consanguinity, genetic counselors can offer carrier testing to individuals. Carrier testing identifies carriers of specific genetic conditions who may not exhibit symptoms but can pass these conditions to their offspring if their partner is also a carrier. By identifying carriers, genetic counselors can guide individuals and couples in making informed reproductive choices. This can involve considering options such as choosing a partner with a different carrier status or exploring assisted reproductive technologies to reduce the risk of having children affected by recessive disorders [38].

Preventive measures: Genetic counseling can also explore preventive measures for individuals and couples at high risk of transmitting recessive disorders. These measures may include pre-implantation genetic diagnosis (PGD), a technique used during in vitro fertilization (IVF) to select embryos without the disease-causing allele, or gamete donation, which involves using donor eggs or sperm to ensure that the offspring do not inherit the deleterious allele. By discussing these options, genetic counselors can assist couples in making choices that align with their preferences and values while minimizing the risk of passing on genetic conditions [39].

Strategies for Reducing Homozygosity

Public awareness and education: Raising public awareness about the implications of increased homozygosity and the potential risks associated with consanguineous marriages is crucial. Education can empower individuals and communities to make informed decisions about family planning. Public awareness campaigns, educational programs, and outreach initiatives can disseminate information about the consequences of increased homozygosity on health and genetics. These efforts can help individuals understand the risks and benefits associated with consanguinity and the importance of considering genetic diversity in family planning decisions [40].

Government policies: Some countries have implemented policies and interventions to reduce consanguineous marriages and the resulting homozygosity. These policies may include financial incentives for marrying unrelated individuals, increasing access to genetic counseling and carrier testing, and encouraging genetic diversity. Government support for these initiatives can help address the potential health risks associated with increased homozygosity and promote family planning strategies that reduce the prevalence of homozygosity-inducing factors [41].

Community support: Community leaders and healthcare providers can be vital in promoting awareness and support for strategies to reduce homozygosity. They can engage in community-based education and support programs to raise awareness of the consequences of increased homozygosity and the importance of genetic diversity. By emphasizing the significance of genetic diversity in the context of family and population health, community leaders and healthcare providers can encourage families and individuals to consider their genetic backgrounds in family planning decisions and seek appropriate genetic counseling and testing when necessary [42].

Ethical and cultural considerations

Ethical Implications of Genetic Counseling

Informed consent: In the context of genetic counseling, informed consent is a fundamental ethical principle. It involves ensuring that individuals or couples are provided with comprehensive information about genetic testing and counseling, including the benefits, risks, and implications of the procedures. Obtaining informed consent is an ethical imperative, as it respects the autonomy of individuals and their right to make decisions about their reproductive health. Informed consent allows individuals to make choices based on a clear understanding of the potential outcomes and the consequences of their decisions, including those related to family planning and genetic testing [43].

Privacy and confidentiality: The ethical principle of privacy and confidentiality is paramount in genetic counseling. Clients often share sensitive genetic information, including their and their family's health history and genetic test results. Genetic counselors must protect this information from unauthorized disclosure. Ethical guidelines stress the importance of maintaining the privacy and confidentiality of genetic information, as it is essential for building trust between clients and healthcare professionals and safeguarding the personal and familial nature of genetic data [44].

Non-directiveness: Genetic counselors are typically encouraged to adopt a non-directive approach. This approach involves providing information, support, and guidance to clients without imposing their values, beliefs, or personal opinions on clients' decision-making processes. The principle of non-directiveness respects the autonomy of individuals, allowing them to make decisions that align with their values and preferences. Genetic counselors should provide information in an unbiased and neutral manner, allowing clients to make choices based on their unique circumstances and values [45].

Cultural Practices and Beliefs

Consanguineous marriages: Cultural practices promoting consanguineous marriages can be deeply ingrained and often have strong roots in social, historical, or religious traditions. To provide effective genetic counseling and support while respecting cultural diversity, it is crucial to understand these cultural norms. Genetic counselors and healthcare professionals should approach individuals and couples seeking counseling with cultural sensitivity and respect. This involves recognizing the significance of these cultural practices to the clients and tailoring counseling to address their specific concerns, including those related to consanguinity and homozygosity [41].

Stigma and discrimination: In some populations with high levels of homozygosity, there may be stigma or discrimination due to misconceptions about the genetic risks associated with consanguinity. Ethical considerations include addressing and challenging such stigma in a culturally sensitive manner. Genetic counselors and healthcare providers have a responsibility to dispel misconceptions and provide accurate information about the risks and benefits of consanguinity. This helps reduce unfounded fears and supports informed decision-making within the cultural context of the clients [46].

Cultural competence: Healthcare professionals and genetic counselors should strive to be culturally competent, which involves understanding the cultural values, beliefs, and practices of their clients. Cultural competence enables effective communication and support while respecting diverse cultural backgrounds. It helps ensure that counseling is delivered in a manner that is culturally sensitive and responsive to the unique needs and preferences of the individuals seeking guidance. Being culturally competent fosters trust and open communication between clients and healthcare providers, enhancing the overall quality of care and support [47].

Balancing Genetic Diversity and Cultural Norms

Cultural sensitivity: Achieving a balance between genetic diversity and cultural norms necessitates a nuanced and culturally sensitive approach. Genetic counselors and healthcare providers must recognize the significance of cultural practices, including consanguineous marriages, within the context of diverse societies. It is essential to respect these cultural norms while addressing the potential health risks associated with such practices. This approach involves open and non-judgmental communication, where healthcare professionals acknowledge the cultural beliefs and practices of their clients while providing accurate information about the genetic consequences of increased homozygosity. By combining cultural sensitivity with genetic expertise, healthcare providers can support individuals and couples in making informed decisions about their reproductive health while respecting their cultural values [48].

Education and awareness: Promoting awareness and education about the genetic implications of increased homozygosity is essential in culturally diverse societies. Providing communities with accurate information empowers individuals to make informed choices about their reproductive health. Educational initiatives should be culturally tailored and accessible to ensure that individuals and communities receive information that aligns with their cultural beliefs and values. This can help dispel misconceptions, challenge stigmas, and foster informed decision-making within the context of cultural diversity [49].

Community engagement: Engaging with communities and involving community leaders is a vital step in bridging the gap between cultural norms and the importance of genetic diversity. Collaboration with community stakeholders, including religious and cultural leaders, can lead to more effective interventions and support mechanisms. Community leaders can advocate for informed decision-making and facilitate open dialogues about the consequences of increased homozygosity and the potential health risks associated with consanguinity. By working together with communities, healthcare providers can create more culturally relevant and impactful interventions that respect cultural norms while promoting the well-being of individuals and families [50].

Future research directions

Emerging Technologies

Genomic sequencing advancements: Future research in the field of homozygosity should leverage the continuous advancements in genomic sequencing technologies. High-throughput sequencing and computational tools have the potential to provide a more in-depth and precise assessment of homozygosity and its effects on the genome. Researchers can conduct large-scale genomic studies to understand the patterns of homozygosity, identify regions of homozygosity associated with specific phenotypes or diseases, and gain insights into the genetic diversity within populations. These advancements in genomics can contribute to a more comprehensive understanding of the impact of homozygosity on human health and evolution [51].

Clustered regularly interspaced short palindromic repeats (CRISPR) and gene editing: The ongoing development of gene editing technologies, such as CRISPR-associated protein 9 (CRISPR-Cas9), offers exciting possibilities for investigating and potentially mitigating the consequences of homozygosity. Researchers can explore the ethical and practical aspects of gene editing to target specific genetic variants associated with recessive disorders in populations with increased homozygosity. This research avenue opens up opportunities to develop strategies for correcting or modifying detrimental alleles, potentially reducing the prevalence of genetic disorders caused by homozygosity [52].

Epigenetics and epitranscriptomics: An emerging area of interest in homozygosity research is the exploration of epigenetics and epitranscriptomics. These fields focus on the study of chemical modifications to DNA and RNA, respectively, which can modulate gene expression and function. Investigating how epigenetic modifications and RNA modifications adapt to increased homozygosity is a promising area of research. These modifications may act as regulatory mechanisms that enable the genome to cope with the genetic constraints imposed by homozygosity. Understanding the interplay between homozygosity and epigenetic or epitranscriptomic modifications can show how the genome adapts to maintain essential functions despite reduced genetic diversity [53].

Longitudinal Studies

Multi-generational studies: Conducting multi-generational studies in populations with varying levels of homozygosity is a vital research direction. Longitudinal studies that span multiple generations can provide valuable insights into the long-term effects of increased homozygosity on fertility, health, and genetic diversity. By tracking changes over time, researchers can gain a more comprehensive understanding of how homozygosity influences population dynamics, genetic adaptation, and the consequences for future generations. These studies can reveal intergenerational patterns of genetic variation and inform strategies for addressing potential challenges associated with increased homozygosity [54].

Health outcomes and aging: Research investigating how increased homozygosity affects health outcomes and aging patterns over a lifetime is significant. Longitudinal studies can provide insights into the relationship between homozygosity and age-related health conditions, such as the prevalence of specific genetic disorders and the impact of genetic diversity on overall health. By examining health trajectories in populations with varying levels of homozygosity, researchers can better understand the long-term implications of genetic homogeneity on health and well-being [55].

Reproductive patterns: Long-term monitoring of reproductive patterns in populations with high levels of homozygosity offers a unique perspective on the evolution of mating preferences, reproductive success, and adaptation to genetic constraints. Research in this area can shed light on how populations adjust their reproductive behaviors in response to increased homozygosity and its potential effects on fertility. Understanding changes in reproductive patterns can help identify adaptive strategies for maintaining genetic diversity and addressing the consequences of consanguinity [56].

Interdisciplinary Research Opportunities

Interdisciplinary collaboration: Interdisciplinary collaboration is pivotal in gaining a comprehensive understanding of the impact of increased homozygosity. Collaborative efforts between geneticists, anthropologists, sociologists, and public health experts can provide a holistic view of this phenomenon. Multidisciplinary research can address the genetic, sociocultural, and public health aspects of increased homozygosity, enabling a more nuanced and comprehensive assessment of its effects on human populations. This approach can offer a more complete perspective on the genetic and societal factors influencing homozygosity and guide the development of effective interventions and policies [57].

Health economics: Research exploring the economic ramifications of increased homozygosity on healthcare systems and society is an interdisciplinary avenue of study. Researchers from fields such as health economics can assess the financial and healthcare burden associated with genetic disorders in consanguineous populations. By analyzing the economic impact of genetic disorders, researchers can provide insights into the cost-effectiveness of genetic counseling, prevention, and healthcare strategies. This research informs healthcare policies and resource allocation to address the healthcare needs of populations with high levels of homozygosity [58].

Psychological and sociocultural research: Understanding the psychological and sociocultural aspects of consanguineous marriages and increased homozygosity is essential. Research in these fields can illuminate the motivations and decision-making processes of individuals and families involved in such unions. Psychologists and sociologists can explore the cultural norms, social pressures, and individual choices that influence consanguineous marriage patterns. This research can provide valuable context for genetic counseling and intervention strategies, as it considers the psychosocial factors that impact family planning decisions and relationships within consanguineous communities [14].

## Conclusions

In conclusion, this comprehensive review has highlighted the intricate relationship between increased homozygosity and human fertility, shedding light on its implications for reproductive health, pregnancy outcomes, and the broader genetic diversity of populations. The findings underscore the heightened risks of inheriting recessive genetic disorders in consanguineous populations and the potential impact on the immune system's diversity and disease susceptibility. Moreover, the review has emphasized the importance of genetic counseling in providing informed support to individuals and couples, particularly in populations with elevated levels of homozygosity. Balancing the ethical considerations of genetic counseling with cultural practices and beliefs remains a critical challenge, requiring a culturally sensitive and community-engaged approach. Looking ahead, further research directions, including the exploration of emerging technologies, longitudinal studies, and interdisciplinary collaboration, are essential to advance our understanding of the implications of increased homozygosity, thus informing the development of effective public health policies and interventions that account for both genetic diversity and cultural norms. Such endeavors are crucial for promoting the well-being and genetic health of future generations in diverse populations worldwide.

## References

[1] (2023). Homozygous. https://www.genome.gov/genetics-glossary/homozygous.

[2] Woods CG, Cox J, Springell K (2006). Quantification of homozygosity in consanguineous individuals with autosomal recessive disease. Am J Hum Genet.

[3] Sharma R, Biedenharn KR, Fedor JM, Agarwal A (2013). Lifestyle factors and reproductive health: taking control of your fertility. Reprod Biol Endocrinol.

[4] (2003). Offspring: Human Fertility Behavior in Biodemographic Perspective. https://pubmed.ncbi.nlm.nih.gov/22675746/.

[5] (2023). The Hardy-Weinberg Principle | Learn Science at Scitable. https://www.nature.com/scitable/knowledge/library/the-hardy-weinberg-principle-13235724/.

[6] Richardson J, Smiseth PT (2023). Chapter two - a behavioral ecology perspective on inbreeding and inbreeding depression. Advances in the Study of Behavior.

[7] Hamamy H (2012). Consanguineous marriages: preconception consultation in primary health care settings. J Community Genet.

[8] (2023). Natural Selection, Genetic Drift, and Gene Flow Do Not Act in Isolation in Natural Populations | Learn Science at Scitable. https://www.nature.com/scitable/knowledge/library/natural-selection-genetic-drift-and-gene-flow-15186648/.

[9] Rudan I (2006). Health effects of human population isolation and admixture. Croat Med J.

[10] Jackson M, Marks L, May GH, Wilson JB (2018). The genetic basis of disease. Essays Biochem.

[11] Sommer S (2005). The importance of immune gene variability (MHC) in evolutionary ecology and conservation. Front Zool.

[12] Spataro N, Rodríguez JA, Navarro A, Bosch E (2017). Properties of human disease genes and the role of genes linked to Mendelian disorders in complex disease aetiology. Hum Mol Genet.

[13] Sharma SK, Kalam MA, Ghosh S, Roy S (2021). Prevalence and determinants of consanguineous marriage and its types in India: evidence from the National Family Health Survey, 2015-2016. J Biosoc Sci.

[14] Iqbal S, Zakar R, Fischer F, Zakar MZ (2022). Consanguineous marriages and their association with women's reproductive health and fertility behavior in Pakistan: secondary data analysis from Demographic and Health Surveys, 1990-2018. BMC Women's Health.

[15] Joly E (2011). The existence of species rests on a metastable equilibrium between inbreeding and outbreeding. An essay on the close relationship between speciation, inbreeding and recessive mutations. Biol Direct.

[16] Gulani A, Weiler T (2023). Genetics, Autosomal Recessive. StatPearls [Internet].

[17] Mathur S, DeWoody JA (2021). Genetic load has potential in large populations but is realized in small inbred populations. Evol Appl.

[18] Chaplin DD (2010). Overview of the immune response. J Allergy Clin Immunol.

[19] Kwiatkowski D (2000). Science, medicine, and the future: susceptibility to infection. BMJ.

[20] (2023). Autoimmune Diseases. National Institute of Environmental Health Sciences. https://www.niehs.nih.gov/health/topics/conditions/autoimmune/index.cfm.

[21] Lvovs D, Favorova OO, Favorov AV (2012). A polygenic approach to the study of polygenic diseases. Acta Naturae.

[22] Phillips PC (2008). Epistasis—the essential role of gene interactions in the structure and evolution of genetic systems. Nat Rev Genet.

[23] Loewe L, Hill WG (2010). The population genetics of mutations: good, bad and indifferent. Philos Trans R Soc Lond B Biol Sci.

[24] Hussain R, Bittles AH (2004). Assessment of association between consanguinity and fertility in Asian populations. J Health Popul Nutr.

[25] Mumtaz G, Nassar AH, Mahfoud Z (2010). Consanguinity: a risk factor for preterm birth at less than 33 weeks' gestation. Am J Epidemiol.

[26] Ben-Omran T, Al Ghanim K, Yavarna T, El Akoum M, Samara M, Chandra P, Al-Dewik N (2020). Effects of consanguinity in a cohort of subjects with certain genetic disorders in Qatar. Mol Genet Genomic Med.

[27] Barrett SC, Charlesworth D (1991). Effects of a change in the level of inbreeding on the genetic load. Nature.

[28] (2006). Genes, Behavior, and the Social Environment: Moving Beyond the Nature/Nurture Debate. Social Environment: Moving Beyond the Nature/Nurture Debate. National Academies Press.

[29] Blue NR, Page JM, Silver RM (2019). Genetic abnormalities and pregnancy loss. Semin Perinatol.

[30] Le Veve A, Burghgraeve N, Genete M (2023). Long-term balancing selection and the genetic load linked to the self-incompatibility locus in Arabidopsis halleri and A. lyrata. Mol Biol Evol.

[31] Tsuang MT, Bar JL, Stone WS, Faraone SV (2004). Gene-environment interactions in mental disorders. World Psychiatry.

[32] Pacheco HA, Rossoni A, Cecchinato A, Peñagaricano F (2023). Identification of runs of homozygosity associated with male fertility in Italian Brown Swiss cattle. Front Genet.

[33] Govindaraj M, Vetriventhan M, Srinivasan M (2015). Importance of genetic diversity assessment in crop plants and its recent advances: an overview of its analytical perspectives. Genet Res Int.

[34] Orr HA (2009). Fitness and its role in evolutionary genetics. Nat Rev Genet.

[35] Koenig D, Hagmann J, Li R (2019). Long-term balancing selection drives evolution of immunity genes in Capsella. eLife.

[36] Anderson JT, Willis JH, Mitchell-Olds T (2011). Evolutionary genetics of plant adaptation. Trends Genet.

[37] Angastiniotis M, Eleftheriou A, Galanello R (2013). Prevention of Thalassaemias and Other Haemoglobin Disorders: Volume 1: Principles. Thalassaemia International Federation.

[38] Read AP, Donnai D (2012). What can be offered to couples at (possibly) increased genetic risk?. J Community Genet.

[39] Hershberger PE, Schoenfeld C, Tur-Kaspa I (2011). Unraveling preimplantation genetic diagnosis for high-risk couples: implications for nurses at the front line of care. Nurs Womens Health.

[40] Joseph N, Pavan KK, Ganapathi K, Apoorva P, Sharma P, Jhamb JA (2015). Health awareness and consequences of consanguineous marriages: a community-based study. J Prim Care Community Health.

[41] Nouri N, Nouri N, Tirgar S, Soleimani E, Yazdani V, Zahedi F, Larijani B (2017). Consanguineous marriages in the genetic counseling centers of Isfahan and the ethical issues of clinical consultations. J Med Ethics Hist Med.

[42] Fatumo S, Chikowore T, Choudhury A, Ayub M, Martin AR, Kuchenbaecker K (2022). A roadmap to increase diversity in genomic studies. Nat Med.

[43] Clarke AJ, Wallgren-Pettersson C (2019). Ethics in genetic counselling. J Community Genet.

[44] Witt MM, Witt MP (2016). Privacy and confidentiality measures in genetic testing and counselling: arguing on genetic exceptionalism again?. J Appl Genet.

[45] Chańska W, Grunt-Mejer K (2022). The directiveness that dare not speak its name. views and attitudes of Polish clinical geneticists toward the nondirectiveness principle. J Bioeth Inq.

[46] Maguire A, Tseliou F, O'Reilly D (2018). Consanguineous marriage and the psychopathology of progeny: a population-wide data linkage study. JAMA Psychiatry.

[47] (2014). Improving Cultural Competence. https://pubmed.ncbi.nlm.nih.gov/25356450/.

[48] Khan N, Benson J, Macleod R, Kingston H (2010). Developing and evaluating a culturally appropriate genetic service for consanguineous South Asian families. J Community Genet.

[49] Bien SA, Wojcik GL, Hodonsky CJ (2019). The future of genomic studies must be globally representative: perspectives from PAGE. Annu Rev Genomics Hum Genet.

[50] Moodley K, Beyer C (2019). Tygerberg research Ubuntu-inspired community engagement model: integrating community engagement into genomic biobanking. Biopreserv Biobank.

[51] Koboldt DC, Steinberg KM, Larson DE, Wilson RK, Mardis ER (2013). The next-generation sequencing revolution and its impact on genomics. Cell.

[52] Uddin F, Rudin CM, Sen T (2020). CRISPR gene therapy: applications, limitations, and implications for the future. Front Oncol.

[53] Hou Q, Wan X (2021). Epigenome and epitranscriptome: potential resources for crop improvement. Int J Mol Sci.

[54] Johnston SE, Chen N, Josephs EB (2022). Taking quantitative genomics into the wild. Proc Biol Sci.

[55] Brooks-Wilson AR (2013). Genetics of healthy aging and longevity. Hum Genet.

[56] Bock J (2002). Introduction: evolutionary theory and the search for a unified theory of fertility. Am J Hum Biol.

[57] Warren JL, Warren JS (2023). The case for understanding interdisciplinary relationships in health care. Ochsner J.

[58] Angevine PD, Berven S (2014). Health economic studies: an introduction to cost-benefit, cost-effectiveness, and cost-utility analyses. Spine (Phila Pa 1976).

